# Health care prioritization process for the elderly in rural Tanzania under decentralized system: Prospects and challenges

**DOI:** 10.1371/journal.pone.0304243

**Published:** 2024-06-10

**Authors:** Malale Tungu, Nathanael Sirili, Gasto Frumence, Alphoncina Kagaigai, Amani Anaeli, Mughwira A. Mwangu, Angwara D. Kiwara

**Affiliations:** Department of Development Studies, School of Public Health and Social Sciences, Muhimbili University of Health and Allied Sciences, Dar es Salaam, Tanzania; Higher Education Partnership / Erasmus University Rotterdam, ETHIOPIA

## Abstract

**Introduction:**

Beginning the early 1990s, many countries globally adopted the third-generation health sector reforms with a focus of strengthening the primary health care system through community participation. On the contrary, three decades later, primary health care systems in many Low- and Middle-Income countries (LMICs) including Tanzania have remained weak. Specifically, priority setting for the vulnerable groups including the elderly have been weak. We aimed to analyse the prospects and challenges of the priority setting process for the elderly health care services following the 1990s health sector reforms in Tanzania.

**Methods:**

We conducted an exploratory case study on priority setting process for the elderly healthcare services in Igunga and Nzega Tanzania. We carried out 24 Key Informant Interviews (KIIs) with the positions of District medical officers, social welfare, Medical Officers in-charge (MOI), planning officers and health system information focal person. Additionally, we carried out two focus group discussions (FGDs), one from each district with six participants from each group. Participants for the FGDs were MOI, health secretary, representative members of Health Facility Governing Committee (HFGC) and Council Health Management Team (CHMT). Data were analyzed using the qualitative content analysis.

**Results:**

Two categories emerged from the analysis of the transcripts. These were the prospects and challenges in priority setting for the elderly population under the decentralized health sector in rural Tanzania. The prospects included; the capacity of the LGAs on priority setting; existence of strategies used by LGAs; availability of teamwork spirit and the existence of guidelines for priority setting at LGAs. The challenges included difficulties of elderly identification, insufficient resources to implement the planned activities at the LGAs, unintegrated digitalized government health information tools or programs at the LGAs, interference of LGAs by the Central Government and low interest of stakeholders on elderly health care.

**Conclusion:**

This study highlights the prospects and challenges facing priority setting for elderly care at the centralized health system in rural Tanzania. From the results the process is well organized but faces some challenges which if not addressed jeopardized and has potential to continue affecting the priority setting. Addressing the challenges highlighted requires joint efforts from both the elderly population in the community, healthcare providers and decision makers across all levels of the health system. This study serves as an eye-opener and calls for a bigger study to get a comprehensive picture of priority setting of the elderly health care in Tanzania.

## Introduction

In the early 1990s, the world embarked on the third generation of health sector reforms [1, 2]. These reforms emphasize strengthening of the primary health care system through strong community participation [3, 4]. Strong community participation is widely recognized as an important strategy in strengthening health systems from a bottom-up approach [5]. However, more than three decades later, most of the primary healthcare systems in many parts of the world have remained weak despite being the entry point to the healthcare system for the majority of the population [6]. Most of the vulnerable groups including the elderly population have been left out in the priority setting process and thus continue to be more disadvantaged [7–10].

Tanzania as for many other parts of the world adopted the 1990s health sector reforms to engage the local community in the priority setting and decision-making for effective and efficient use of resources [3, 10]. These reforms involved organizational, managerial, ideological and financial reforms (Proposal for health sector reforms 1994). Organizational and managerial reforms made the district to be the focal point for planning and implementation of all health programs following the adoption of decentralization by devolution health system administration [6, 11]. Decentralization is the transfer of power, authority, functions and service delivery from the central to local authorities. Decentralization by devolution means the legal transfer of power to the elected local political organs of local government authorities (LGAs) democratically from the central government with a defined set of functions [3, 6, 12].

The reforms in Tanzania, like in other parts of the world especially in Low and Middle-Income Countries (LMICs), aimed to enhance people’s well-being through improving access to health services by bringing health services closer to the people including vulnerable groups. However, the elderly population has continued to succumb to limited access to quality healthcare services in the primary healthcare system in Tanzania [10]. Since it is difficult to provide all possible health care services for all people, each health system must set priorities by considering what will be provided and what will not be provided. Priority-setting entails identifying systematic rules to decide on the distribution of limited health care resources among competing programs or patients. It occurs at all levels of every healthcare system and is one of the most important issues in healthcare management today [13].

In 2018, Tanzania introduced a direct health facility financing (DHFF) program that aimed at improving the corresponding payment to priority areas, enhancement of transparency and accountability, proper management of funds and high-quality service delivery [14]. The program was introduced to increase accountability and governance in the health system at the primary healthcare level and to increase health system responsiveness for patients who receive healthcare in the respective health facilities including vulnerable groups like the elderly population. The introduction of DHFF replaced the system of channeling the financial resources through the council level to the primary healthcare facilities which led to delay in accessing the finances by the health facilities [14–16].

Priority setting in health systems in Low- and Middle-Income Countries (LMICs) like Tanzania is important in ensuring proper allocation of scarce resources for the provision of responsive health services. By definition, priority setting in health care means the process of choosing among different alternative health programs and services by ranking the alternatives by normative and technical rules that can lead to determining the minimum or basic package of health care services [17]. Proper priority setting must ensure financial protection to vulnerable groups, for instance, the elderly population. Therefore, the process of priority setting must be inclusive of these community members. In Tanzania, public involvement is considered a significant dimension in healthcare planning and priority setting for decision making within decentralized health systems across the country.

Priority-setting is one among many challenges facing health decision-makers for elderly health care services in Tanzania. There is a mismatch between the need for healthcare and the availability of resources for the elderly. The elderly face many health problems [8] and challenges in accessing healthcare services [18] which need to have an improved prioritization process. Priority-setting needs framing organized rules to decide on the distribution of the limited health care service resources for the elderly [13]. This process of explicitly deciding on values can likely be facilitated by purposive research specifically to the health workers/managers and decision-makers at the local government (district level) by providing their experience in setting priorities for the health care services of the elderly. Most of the elderly suffer from different diseases, especially in rural areas.

Studies on priority setting at the district level are scanty and not specific to the healthcare services for the elderly. Most of them are based on the general priority setting process against accountability for reasonableness from the perspectives of the CHMT, local government officials, health workforce and committees, actors and contextual factors shaping decentralised healthcare priority setting and other issues which are not specific to the elderly health care priority setting [13, 19–23]. This study, therefore, sought to analyse the prospects and challenges of the priority setting process for the elderly health care services following the 1990s health sector reforms in Tanzania.

## Methods

### Study design

This study adopted an exploratory case study design that used Key Informants Interviews (KIIs) and focus group discussions (FGDs) using the interview guide. The questions in the interview guide were constructed from reviewing relevant documents and literature on the implementation of priority setting at the district level. An exploratory qualitative study was appropriate for this study to explore the information on current methods and procedures used to set priorities for health services of the elderly from the KIs who participate in priority setting and decision making at the district level.

### Study context

This study was conducted in Nzega and Igunga districts in Tabora Region of western Tanzania between July and August 2020. The decentralized health system has different levels in making decisions and setting priorities at LGAs. These levels start from the lower level to the national level including community, facility, district, regional and national levels. At the district level, each council is required to prepare a CCHP each year to access funds allocated by the central government. The process of preparing CCHP is done by the CHPT composed of CHMT members and other representatives from communities, private and public health providers and Non-Government Organizations (NGOs). This study involved the representatives of the planning team at different levels of each district. These include representatives from CHMT, Health Facility Governing Committee (HFGC), Council Health Service Board (CHSB) and the full council. The provision of health services at the district level is composed of district hospitals, health centres and dispensaries. Among the total population of the two districts, the number of elderly people is approximately 50,547 which is 5% of the total population [24]. The Nzega and Igunga districts were appropriate places for this study because most elderly people (around 92%) live in rural areas. Furthermore, the two districts were among the first districts to implement Community Health Fund, with Igunga being the first starting in 1996. Both primary and secondary health facilities are available and offer health care services to the elderly in these districts and thus we found them appropriate as priority setting can view the organization of health facilities from primary to secondary and beyond. Administratively, Nzega district is divided into two councils (town and rural council) that are divided into 37 wards with a total of 151 villages/streets, and Igunga has one council that is divided into 26 wards with a total of 93 villages/streets.

### Data collection

Data collection was done between July and August 2020. We started by reviewing the documents to familiarize ourselves with the decision-makers who are involved in priority setting and planning. From reviewing the documents, we arrived with 24 KIIs for the study who were purposefully selected based on their day to day roles on priority setting for the elderly care.

After attaining information saturation of most of the categories, meaning that there was no new information that was coming with additional interviews data collection stopped at 24 respondents. This was because the aim was to explore information on the implementation of the decentralized health system procedures during setting priorities. The interview guide was used to explore information on the process of priority setting for the elderly. Respondents were asked about the importance of LGAs, their experience with the procedures used for priority setting, identifying the elderly for granting the exemption, challenges and their recommendations to have improved health services among the elderly. An in-depth interview was the main approach used to collect information from the respondents. Four researchers (whereby two were faculty members from Muhimbili University of Health and Allied Sciences (MUHAS) and two were Master’s Graduates from MUHAS) conducted the interviews. The faculty members facilitated the interview while others facilitated the audio recording of the interviews, taking notes on the key themes, asking additional questions and monitoring any interaction. The interviews lasted for 50–74 minutes. We also conducted one FGD from each district on exploring information on the process of priority setting. The FGDs which included 2 HFGC members, 2 CHMT members, MOI, and HS from each district were conducted in one of the rooms at the district council offices. Each FGDs included 6 respondents and were selected from the district hospitals from each district. The FGDs lasted between one and two hours and were audio-recorded using a digital audio recorder. Other research assistants took notes during the interview.

### Data analysis

The audio-recorded interviews (conducted in Kiswahili to facilitate communication) were reviewed and transcribed verbatim from audio to written documents then translated into English using the qualified translators for analysis and extraction of quotes. Data were analyzed using the content analysis approach. This approach is used to determine the presence of concepts within texts or a set of texts which limit bias. The approach helped to develop categories from the text data inductively for capturing the experiences of the participants. In addition, this approach entails the interpretation of the content of text data through a systematic classification process of coding and identifying themes or patterns [25, 26]. The transcribed data, field notes and documents were carefully reviewed and read to identify broad areas to form initial codes and codes. The authors extracted primary codes, discussed, revisited and final codes agreed by all authors. Similar codes were grouped together through comparing codes to have subcategories. Subcategories were further analyzed to differentiate their similarities and differences. Similar subcategories with related concepts were grouped to form categories.

### Ethics approval and consent to participate

Ethical clearance was obtained from the Muhimbili University of Health and Allied Sciences (MUHAS research review board) in June 2020 (MUHAS-REC-6-2020-288). Permission for data collection in Tabora region was granted by the Regional Administrative Secretary. Permission for data collection was granted by the District Executive Directors of the Igunga and Nzega districts. Participants were duly informed of the purpose of the study and their rights. Written informed consent for this study that includes data collection and consents to participate was requested and obtained from the participants and they were assured of their anonymity in publications. All methods were carried out in accordance with relevant guidelines and regulations of the approval bodies and in accordance with the Declaration of Helsinki.

## Results

Two major categories emanated from the analysis of the KIIs and FGDs. These are prospects and challenges for priority setting in rural Tanzania. The prospects included; the capacity of the LGAs on priority setting; existence of strategies used by LGAs; availability of teamwork spirit and the existence of guidelines for priority setting at LGAs. The challenges included difficulties of elderly identification, insufficient resources to implement the planned activities at the LGAs, unintegrated digitalized government health information tools or programs at the LGAs, interference of LGAs by the Central Government and low interest of stakeholders on elderly health care. It involved 24 (7 female and 17 male) respondents who are involved in making a decision, planning a budget and setting priorities at the district level. These were serving at different positions to include District medical officers, Social welfare, Medical Officers in-charge, planning officers and health system information focal person‥ For each district we purposively selected 1 District Planning Officer (DPLO), 1 District/Town council Medical Officer (DMO/TMO), 1 Medical Officer In-charge (MOI), 1 Health Management Information System (HMIS) focal person, 1 District Social Welfare Officer (DSWO), 2 HFGC members, 1 District Health Secretary (DHS), 1 Hospital Secretary (HS), 1 CHSB members and 2 CHMT were invited to the study making a total of 24 KIs.

### Prospects for priority setting for the elderly

#### The capacity of the LGAs on priority setting for the elderly

This study found that the LGAs can conduct a priority-setting process that involves different stakeholders including experts from the LGAs (CHMT), community (through village meetings, HFGC), local leaders (hamlets, village executive officer (VEO) & ward executive officer (WEO)), representative elderly at the elderly council, NGOs and other stakeholders. The challenges of the capacity of the LGAs include; a lack of well-trained personnel who are involved in planning and setting priorities and insufficiency/scarcity of resources that limit the fulfillment of the planned priorities.

“…we do not have a formal training about taking care and setting priorities for elderly but we normally use experience, guidelines from the ministry of health and directives for the leaders…” (KI #2).

#### Existence of strategies used by LGAs to prioritize elderly

Strategies for prioritizing the elderly at LGAs include; the issue of dealing with their complaints (through council elderly, DSWO), prioritizing poorer elderly than those with HI and those who can pay for health services, identifying the elderly and providing IDs for exemption purposes. Some elderly are put at different camps where the LGAs are responsible to provide care. In addition, LGAs through DSWO and local leaders provide education and awareness about elderly matters to the community and health workers. The use of different government information systems including the Government of Tanzania ‐ Hospital Management Information System (GoT-HoMIS), direct health facility financing (DHFF), planning and reporting database (PlanRep), Facility Financial Accounting and Reporting System (FFARS), etc helps to control the resources from the central government and own sources which can increase the provision of health services among the elderly.

“Since LGAs are located near to the local people including the elderly in rural areas, they are aware of the elderly problems that can be easier for them to arrange/set priorities for the best health services for the elderly. In short, LGAs play a great role and have a chance to benefit the elderly in rural areas” (KI #4).

#### Presence of teamwork spirit of planning and priority setting for the elderly at LGAs

Planning and priority setting process for the elderly at LGAs is implemented on a teamwork basis. This teamwork involves experts from the council level, community through general meetings at the village or hamlet level and HFGC representatives, Local leaders (hamlets, VEO, WEO), elderly councils, and private sectors on the provision of health services whereby some of the private health facilities provide health services to the elderly in collaboration with the government under public-private partnership (PPP). In addition, other stakeholders who are in priority settings include NGOs, development partners and politicians.

“Aah… we work as a team…where we use experience through discussion. During setting priorities every individual will provide his/her own opinions…. This is to say elderly matters…of course, even the government (through its regional commissioner) declares that the elderly should be prioritized” (KI #1).

#### Existence of guidelines for priority setting at LGAs

Our results show the existence of different guidelines which are prepared by the central government to help the process of priority at LGAs. This includes the 13 priorities at LGAs whereby all activities which are supposed to be planned and prioritized should base on these priorities which are set by the central government. Elderly matters are included in the social protection under the social welfare office.

“Using the available guideline, under LGAs we have 13 national priorities. Among these priorities, a priority for the elderly is not included. It is included under the social welfare office. Under social welfare office deals with people living with disabilities and others including elderly” (KI #5).

Other guidelines underlined by the KI are CCHP and O&OD which provide a clear process of priority setting. This priority setting process involves the following stages: situation analysis, extracting data from situation analysis, analyzing the obtained data, determining the extent of the problem, suggesting which intervention to be taken to solve the existing problem and then before implementation of any intervention needs to consider the available resources.

#### Existence of the exemption policy for elderly

Results of this study show that all elderly are eligible for accessing free health services specifically to the public health facilities. Therefore, exemption or waiver to the elderly starts from those 60 years old and above who are eligible for free health services. LGAs bear the responsibility of making sure that all elderly who are eligible should access free health services. The impact of this is an increase in access to health services among the elderly which leads to the burden of an increase of health facility expenditure compared to the collected revenue. In addition, a little awareness of this policy among the health workers, some of the decision-makers who are involved in priority setting, the elderly and the community.

“The burden of the exemption to the health facility still not known who covers the costs. DHFF have no category which covers the costs of exemption. In one way or another, the gap between exemption and revenue is reduced by ordering medics who at least cover those with exemption from MSD. Complaints about elderly healthcare services in rural areas, some of the healthcare providers to some health facilities (HF) refuse to serve the elderly for free, they are forced to pay even though they are eligible to be exempted” (KI #7).

### Challenges for priority setting

#### Difficulties of elderly identification

Participants stated that the process of identifying the elderly for the provision of IDs to access health services from the villages was difficult. They added that most people had no birth certificates to confirm their ages and thus it was difficult to ascertain those who were aged above 60 years as per criteria.

“…during identification process of the elderly to have an exemption ID, they are required to have birth certificates to get an identification letter from the hamlet or VEO which confirms their ages whereby most of the elderly in villages they do not have birth certificates which make the process to be difficult…” (KI #1).

#### Lack of clarity of the exemption policy

Lack of clarity of the exemption policy was stated as a challenge to priority setting by majority of the participants. They stated that it was not clearly stated as to what should be exempted and not exempted to service recipients. The criteria of age was also debated to be adequate for exemption to the elderly.

#### Insufficient resources to implement the planned activities at the LGAs

KIs informed the researchers that there is a huge difference between resources allocated and what is planned for health services of the elderly. They added that own sources from the LGAs and funds from the central government have no specific percentage devoted to the elderly health care services and they are generally inadequate.

“…there are many sources from the council that have been taken by the central government that leave the council with not enough funds to finance different programs…” (KI #7).

#### Unintegrated digitalized government health information tools or programs at the LGAs

Informants of this study stated that the government digitalized health tools are not well integrated whereby most of them do not help direct the elderly. For instance, GoT-HOMIS which is the government digitalized health information system that records all transactions of all patients at the health facility but does not capture exempted elderly.

“…in the council which I lead we have decided to install GoT-HOMIS system to all health facilities which have access to electricity, especially on the section of cash and receipts. The system can track patients’ records including money paid at the health facility …” (KI #6).

#### No specific law which can protect the implementation of the exemption policy for the elderly

At the LGAs there is no specific law/by-law that protects the interest of the elderly. This was also advised by one of the respondents as follows; “…I advise that the process of initiating a strict law that will protect elderly should be done immediately…” (KI #10). There is no specific law that can be protected the exemption policy from its implementation to individuals who can violate that policy whether the health workers or the community.

#### Interference of LGAs by the central government

Interference from the central government was stated to contradict the aim of decentralization which was to give the LGAs autonomy in decision making. They added that some of the LGA’s sources of revenue have been taken by the central government contrary to the aim of decentralization by devolution. Furthermore, participants stated that the LGAs were directed by the central government to provide free health services to the elderly while their sources of revenue had been taken.

“…we are not free as has been described that LGAs have an autonomy to do their things…some of the source of revenue at council level have been taken to the central government …” (KI #7).

#### Low interest of stakeholders on elderly health care

Participants felt that most of the stakeholders including NGOs, MPs and development partners were not interested on the elderly matters. They added that stakeholders are interested and are investing in HIV/AIDS, pregnant women, children under five, specific diseases like TB, and malaria.

“…we have CHWs but deal with pregnant women, mothers and children…. Most of the NGOs are for HIV but not the elderly…also politicians like MPs I never experienced them engaging themselves in helping the elderly…” (KI #3).

## Discussion

This study aimed to analyse the prospects and challenges of the priority setting process for the elderly health care services following the 1990s health sector reforms in Tanzania. The study has highlighted the capacity of the LGAs on priority setting; existence of strategies used by LGAs; availability of teamwork spirit and the existence of guidelines for priority setting at LGAs as prospects for priority setting for the elderly healthcare at the decentralized health system. The study has also uplifted difficulties of elderly identification, insufficient resources to implement the planned activities at the LGAs, unintegrated digitalized government health information tools or programs at the LGAs, interference of LGAs by the Central Government and low interest of stakeholders on elderly health care as challenges facing priority setting for the elderly healthcare at the decentralized health system.

### Prospects on the priority setting for the elderly

As the findings revealed by this study, the LGAs have the capacity to conduct a priority-setting process since they involve different procedures from the local level to the district level and involve different stakeholders at each stage. At the local level involving the community (through village meetings, HFGC), local leaders (hamlets, VEO & WEO), and representative elderly at the elderly council. At the district level involve experts from the LGAs (CHMT), NGOs and other stakeholders. This study has also indicated a lack of well-trained personnel who are involved in planning and setting priorities and insufficiency/scarcity of resources which limit the fulfilment of the planned priorities. The findings concur with other studies done in Tanzania and elsewhere [10, 27–29] which reveal the challenges facing LGAs on the capacity of the personnel in setting priorities and planning. The findings indicate that there are different strategies used by LGAs in priority settings like dealing with elderly’s complaints, prioritizing the poorer and with no HI than rich and those with HI and provision of education/awareness to the community and health workers about the elderly. The findings of this study have also revealed that priority setting at LGAs is teamwork which involves different stakeholders and the use of different guidelines on priority setting. Regardless of its challenges, the implementation of the exemption policy has helped to increase access to health services among the elderly.

### The challenges facing priority setting at LGAs

One of the findings of this study is on the process of identifying the elderly who are eligible for IDs to access free health services. The challenges in this process include difficulties in identifying the elderly’s age, difficulty to predict the number of elderly per year during budgeting and insufficient budget to provide IDs to every elderly. Most elderly in rural areas have no birth certificates to confirm their ages which results in difficulty to access health care services using the exemption. These findings concur with other studies from Tanzania including the National Ageing Policy [10, 30–32] revealing that one of the problems facing the elderly to be allowed to access free health services is difficulties in proving their ages to be eligible for the exemption and waiver health services.

Another challenge facing the elderly which was identified by the KIs is that there is lack of clarity of the exemption and waiver policy. Findings indicate that this policy applies to public health facilities and only a few private health facilities. The private health facilities which offer waivers and exemptions are those that receive funds from the government. This is because there is no direct compensation of funds for the exemption and waiver at the health facility from the central government specifically for the elderly. The burden of unfunded health services offered at the health facilities for the elderly as exemptions and waivers have increased [33]. In addition to that, findings indicate that the policy is still controversial among health workers, policymakers and the elderly themselves. This led to the difficulties to have a uniform implementation of this policy since every health worker at different health facilities has their definition of the policy because there is no specific law and guideline that can define the policy. The insufficient resources also limit the fulfilment of the planned activities for the elderly. These results concur with other studies done in Tanzania that the policy is not effective due to confusion about the policy on the eligibility criteria to access free health services [34–36].

Another challenge with this policy which was revealed during the study was that there is no specific law governing this policy which protects the elderly’s interests. The National Ageing Policy and other studies [30, 32, 34] explained the establishment of law by the central government to be implemented by the LGAs to protect the elderly’s interests. However, this law is still in the planning process and has not been established [10, 31, 37, 38]. This has led to the unprotected elderly’s interest in its implementation by individuals (whether it is the health workers or community members) who can violate that policy.

Findings indicate that at LGAs, the health information systems are not well integrated. This is because the health information systems/programs and guidelines are not well coordinated to solve the elderly’s problems to have improved health care services. These government health information systems/programs and guidelines include GoT-HOMIS, DHFF, FFARS, CCHP, PlanRep and 13 national priorities at LGAs do not touch directly with the elderly. All these health information systems/programs are applied at the LGAs and they can help the free access to health services. For instance, GoT-HOMIS is the health information system that records all transactions of all patients at the health facility. This can be used to extract the information about exemptions and waivers of the elderly at the health facility which can be repaid from either the central government or LGAs. DHFF is the system that reimburses funds for all activities planned at the health facility. This system doesn’t help direct the elderly since activities that favour the elderly are done at the council level like IDs and the identification process of the elderly and not at the health facility which receives funds directly from the central government. Another challenge with this decentralized health system is delays in reimbursing funds from the central government to the health facilities which affects some services which can help the elderly. In addition, the CCHP guideline indicates the percentage of the budget for each activity but not for the elderly. These findings concur with other studies in Tanzania and elsewhere about the challenges of these decentralized health systems which reveal the delay of reimbursing funds and uncoordinated systems [14, 15].

The findings of this study revealed that the LGAs were supposed to have autonomy from the central government. However, the central government interfere with so many issues in setting priorities which led to the underperforming of LGAs activities. For instance, some of the own sources which were collected by the LGAs have been taken by the central government which creates dependence. District councils are instructed with directives by the central government to provide free health services to the elderly but no funds are provided for the free services to the elderly. DHFF have no category which covers the costs of exemption from the central government for the elderly at the health facility.

The National Ageing Policy explained clearly the importance of the stakeholders to be involved in setting priorities and planning for the elderly. The challenge with this strategy is that most of the stakeholders including NGOs, MPs, development partners, etc. have law interest to the elderly health care compared to HIV/AIDS, pregnant women, children under five years old, and specific diseases like TB and malaria. CHWs are not well utilized to help the elderly due to financial problems at the LGAs. These CHWs are not responsible for the elderly, they are responsible for children (under 5 years old) and mothers or pregnant women, although they can be assigned to the elderly on the outreach services. MPs are not much involved in helping the elderly. Elderly matters are not much prioritized at all levels including family level, village level, ward and district council level.

### Strength and limitations of the study

The strength of this study is that it has provided valuable information that will contribute toward improved methods and procedures for priority setting to improve the provision of healthcare services to the elderly. The study also revealed the challenges facing the planning team and LGAs during setting priorities for improving healthcare services for the elderly. Since the study was conducted among people who are responsible for policymaking at the LGAs, the limitation of the study may be based on the fact that such respondents are likely to be defending the ways they run things. They may describe the methods and procedures for priority setting to defend themselves. However, the findings of the study provided an understanding of the procedures and methods for priority setting to have improved healthcare services for the elderly. To address the limitations the study included many participants including members of the different committees, whereby most of them are part of the community.

## Conclusion and recommendations

This study indicates the importance of LGAs on the implementation of priority setting process to have improved health care services under decentralized health system in many developing countries including Tanzania. However, the study encountered some challenges on the implementation of priority setting process at district level include difficulties in identifying the elderly’s age to be eligible for the exemption purposes, lack of clarity of the exemption policy to the elderly and health care workers, there is no specific law governing this policy which protects the elderly’s interests, the health information systems are not well integrated to help the priority setting process for the elderly and the LGAs were supposed to have autonomy from the central government in regards to mobilization of resources and setting their priorities according to the needs of the specific district or council. Therefore, this study will inform decision makers on the importance, prospects and challenges of the priority setting process under decentralised health systems for providing healthcare services to the elderly in countries with scarce resources like Tanzania.

This study has come up with the following recommendations that were gathered during the KIIs: First, is the enactment of a specific law that will protect the elderly’s interests. The National Ageing Policy, HelpAge International [39–41] and other elderly day celebrations have recommended enacting a law that will specifically protect the elderly. Second, having a specific per cent of the LGAs revenue which should go to the elderly health services (elderly matters). Third, to build capacity among the planning team who are involved in the planning and priority setting process to be aware of the elderly problems. Fourth, improvement of the elderly identification process by having a database at the LGAs level and should actively involve the CHWs in the process. Fifth, sensitization and awareness building among stakeholders including the elderly themselves, the community, health workers, TASAF, and NGOs, on the special needs of the elderly people and how to help them. Sixth, to encourage the elderly who have health insurance to use them at the health facilities instead of opting for free services under the exemption system. Finally, the central government or LGAs should repay the exempted fees to the health facility or provide them with HI.

## Supporting information

S1 File Content analysis process. (DOCX)

## List of abbreviations

CCHP: Comprehensive Council Health Plan
CHF: Community Health Funds
CHMT: Council Health Management Team
CHPT: Council Health Planning Team
CHSB: Council Health Services Board
CHWs: Community Health Workers
DHFF: Direct Health Facility Financing
DHS: District Health Secretary
DMO/TMO: District/Town council Medical Officer
DPLO: District Planning Officer
DSWO: District Social Welfare Officer
FFARS: Facility Financial Accounting and Reporting System
FGDs: Focus Group Discussions
GoT-HOMIS: Government of Tanzania ‐ Hospital Management Information System
HFGC: Health Facility Governing Committee
HMIS: Health Management Information System
IDs: Identities
KIIs: Key Informant Interviews
LGAs: local government authorities
LMICs: Low- and Middle-Income Countries
MoH: Ministry of Health
MOI: Medical Officer In-charge
NGOs: Non-Government Organizations
NHIF: National Health Insurance Fund
O&OD: Opportunities and Obstacles for Development
PlanRep: Planning and Reporting database
PO-RALG: President’s Office, Regional Administration and Local Government Tanzania
PPP: Public-Private Partnership
QALYs: Quality-Adjusted Life Years
VEO: Village Executive Officer
WEO: Ward Executive Officer
