# Supplementary material for: Health care prioritization process for the elderly in rural Tanzania under decentralized system: Prospects and challenges
Source: PLoS One. 2024 Jun 10;19(6):e0304243. doi: 10.1371/journal.pone.0304243 (PMC11164382; doi:10.1371/journal.pone.0304243)
Supplement: S1 File — (DOCX) [file pone.0304243.s001.docx]

**Content Analysis process**

To analyse the current methods and procedures for priority setting on the healthcare services of the elderly in rural areas.

| **S/N** | **Broad area** | **Initial codes** | **Codes** | **Sub-categories** | **categories** |
| --- | --- | --- | --- | --- | --- |
| 1 | Priority Setting process | - Existence of planning team CHMT, CHGT, etc. (Role distribution) - Identification of the elderly - Existence of SWO who lead to identification of the elderly - LGAs help on setting priorities using local leaders from lower level (village) to the higher level (district council level) – DSWO 20 - Identification of the elderly before being given IDs starts from village leaders where they identify names and submit to the social welfare department for provision of IDs (pg4) - Village/hamlet leaders approve the age of the elderly - Provision of the IDs to the elderly - Special window for the elderly at the HF (doctor’s room and medics window) - First priority for the elderly during budgeting on each year is the budget for IDs (to give IDs)-DPLO 1 - During allocation of funds where prioritize essential services including medicine for the elderly – DHFF 12 - Most of the consideration about elderly with DHFF system is to put more priorities on the services that touches elderly as well like medicine, test kit of the chronic diseases –DHFF 13 - Guideline which indicates what to be followed during priority setting - DHFF focal person involved on priority setting process indirectly especially during budgeting i.e. budget for provision of IDs (the number of elderly against the number of IDs regarding the available budget), also to estimate the cost of each ID – DHFF 1 - There is no specific category for the elderly among 13 priorities at the LGAs. Elderly are included at the social welfare which have other groups like peole living with disabilities, etc. – TMO   -Elderly are more prioritized than young age  -if more rooms are available at the health facility, elderly are prioritized by being given their own consultation and pharmacy rooms  -If no enough rooms the elderly will be given first priority in the queue, excluding the those with serious condition   - There is no special training to deal with elderly issues and how to prioritize, they use experience and guidance from the ministry of health - TMO - Priority setting is a process which starts from lower level to district level (TSWO-Ig) | - Existence of planning team - Partnership with village leaders in identification of elderly - Involvement of multiple stakeholders in setting priorities - Provision of special IDs to the elderly - Existence of special window for the elderly in health facility - Allocating budget for the elderly healthcare services - Existence of guideline for priority setting - Existence of 13 priorities at the LGAs | - Capacity for planning (human and nonhuman resources) =how - Implementation strategies for prioritizing elderly=what - Planning as a team work activity=who - Guidelines for priority setting (13 priorities) | - The capacity of the LGAs on priority setting - Experts - Training - The r/ship btn dd vs ss - Strategies which are used at LGAs to prioritize elderly and other disadvantaged groups - Dealing with complaints - Prioritizing more poor than those have HI and who can pay for health services - Special department at LGAs dealing with elderly matters - Some elderly are put at different camps - Awareness about elderly matters (health workers, community, decision makers, elderly themselves) - Using different government systems (GoT-HoMIS, DHFF, PlanRep, FFARS, ect) - Priority setting as a teamwork at LGAs - Involves different expects - Community involvement - Local leaders - Different committees - Elderly councils - Involvement of private sectors on provision of health services (PPP) - Involve different stakeholders - Guidelines prepared by the central government helps the process of priority at LGAs - 13 priorities - A clear process of priority setting - Support from the central government during priority setting at LGAs - Exemption/waiver policy - Helps to relief elderly from paying health services (free services) - Its challenges include(its burden to the HF, controversial on its implementation, awareness among health workers about the policy, etc.)      - Perception about elderly agenda - Different perceptions from community, family members, experts, decision makers - Bad/good perception - Positive/negative view about elderly agenda - Moral issues among family members about the elderly - Challenges during planning and priority setting - Exemption/waivers is not clear to the elderly themselves, experts, health workers, etc. - No specific % of the budget from the LGAs - Scarcity of resources - Unintegrated health systems at LGAs which doesn’t help elderly direct - No strict law which can protect elderly matters - The burden of exemptions vs the available resources - Autonomous of LGAs is still questionable whereby still there is an interference of central government |
|  |  |  |  |  |  |
|  |  |  |  |  |  |
|  |  |  |  |  |  |
|  |  |  |  |  |  |
|  |  |  |  |  |  |
| 2 | Priority setting practices/experiences | - Acceptability of issues related to elderly in meetings is positive and is discussed in a positive way (pg4) - The process of budgeting starts from CHMT, pass through the health board, then to the committee of social services, then to the financial committee and finally to the full council at district council level whereby at each step should consider the vulnerable groups including elderly - DPLO is for planning and budgeting at district level – DPLO 2 - During setting priorities elderly issue is an agenda mostly presented by social welfare department - In one way or another, the gap between exemption and revenue is reduced by ordering medics which at least covers those with exemption from MSD – DHFF 8 - Referral with exemptions can be acceptable to another hospital only if the elderly have an ID or any identification from local leaders but not sure for the chronic diseases – DHFF 9 - Priority setting is done according to the extent of the problem by considering the available resources-TMO - More resources have been allocated to the more serious problem –TMO - More health workers and with high skills are allocated most to where the work load is too much –TMO - Priority setting is not done depending on old age, but on the type of diseases that occur frequently in this age group – TMO   9. Priory setting at the facility level (sera).  -Elderly are more prioritized than young age  -if more rooms are available at the health facility, elderly are prioritized by being given their own consultation and pharmacy rooms  -If no enough rooms the elderly will be given first priority in the queue, excluding the those with serious condition  Display of elderly policy on the notice board at the health facilities   - Elderly policy education to all people at the health facilities during the morning sessions. – TMO   Special window (doctor/pharmacy) for the elderly (MOI-Ig).  Set a special day for checkup of the NCDs (Thursday) for the elderly  Exemption process through social welfare office  To set a budget for the medicines which are most helpful to the elderly   - Promoting awareness through frequent presentations about elderly health care services at the health facility to the health workers. - LGAs have direct benefits to the priority setting process due to the fact that they are close to the elderly people. – TMO - Priority setting process;   Situation analysis from previous/present year  Get data from situation analysis  Analyse the data  Determine the extent of the problem  Which intervention to take   - Consider the available resources (Human resources, money, infrastructure etc) – TMO - Responsible office at council level for elderly is SWO department which is involved on the preparation and planning a budget for the elderly, help elderly to get waiver/exemption, etc. - The decentralized health systems has efficiency and effectiveness on priority setting process which starts from lower level (village/hamlet/mtaa) to the high level (district). Also helps to identify the problems from the lower level because has a direct contact with the community. - Sometimes in order things to move we take our own money to help elderly (TSWO-Ig). | - Consideration of elderly during priority setting practices - Budgeting is the cascade process - Importance of LGAs during priority setting - Existence of waiver and exemption for the elderly - Problem identification (diseases) during setting priorities and allocation of funds - Existence of public notification about elderly policy (sera) at the HF - Existence of the department for handling elderly’s complaints. - Raising awareness to the community about elderly matters. - Stages of priority setting | - Balancing between the demand and available resources during budgeting. - Existence of waivers/exemptions to protect the vulnerable elderly. - Engaging the community in understanding that elderly are more prioritized. - Dealing with elderly complaints is a priority - Existing of a clear process for priority setting. |  |
|  |  |  |  |  |  |
|  |  |  |  |  |  |
|  |  |  |  |  |  |
| 3 | Healthcare services for the elderly in rural area | - Posters alerting everyone to prioritize elderly at the health facility helps elderly to reduce the waiting hours. - Priority on the elderly matters starts from the health facility with the slogan of “Elderly first” - DHFF 24 - The office to present their (elderly) complains is DSWO and MOI – DHFF 24 - The central government cover the costs of free services of the elderly through sending medicines direct to the HF(pg5) - The exemption gap most of the time is refilled by the government through medicines and is not refilled 100% which is therefore still a challenge DPLO 14 - Elderly with 60+ and living in poor condition are more prioritized - Medications are bought by considering aid distribution, top ten diseases and through discussion with the elderly councils –TMO   Education has been provided to all by explaining the eligibility of age for exemption   - Identification of the elderly aged 60+ by providing them IDs or letter from local leaders - Through the guideline, all elderly aged 60+ should be provided IDs for exemption but due to the scarcity of resources: - Elderly with HI are excluded from this service - Elderly living in a poor condition are more prioritized - Elderly with the ability to pay for health services are excluded - The main factor which simplify the identification process of the eligible elderly for exemption is through local leaders who make follow-up to know the status of the elderly (poor, rich, age, HI, etc) (TSWO-Ig). - FBOs health facility have been funded by the government some amount for the compensation of the exemption of the disadvantaged groups including elderly, <5 years, pregnant women, etc. | - Existence of support from the central government about elderly healthcare services - Existences of the guideline for elderly identification process - Exclusion of some elderly on accessing free health care services - Involvement of the private sector on provision of health care services to the disadvantaged groups including elderly. | - Central government support - Exclusion of some elderly on accessing free health care services - Existence of Public Private Partnership on provision of some of the health care services among the elderly. |  |
|  |  |  |  |  |  |
|  |  |  |  |  |  |
|  |  |  |  |  |  |
| 4 | Elderly population | - Elderly, is 60+ years old - elderly councils-pg11 - Social welfare department represents the elderly during setting priorities and budgeting (pg12), also solves elderly problems and complains at the health facility and family members DPLO 10 - Some of the complaints about elderly been represented by the council representatives (councilors) during the meetings – DPLO 11 - Elderly councils are only found in few places – DPLO 32 - Elderly council made up with elderly from village level to district council level, and there a guideline that guides how to run those elderly councils - Problems facing elderly - BPH - Diabetes - Tezi dume (prostate cancer) - Urinary system - Pneumonia/kifua kubana - Pressure - heart disease - Body pain - Kisukari - Cough/kukohoa - Malaria   Complaints from the elderly about access to health services;  Difficult to identify age, they complaint esp if they don’t have IDs to identify their age for exemption  The same complaint comes from the councilors  No medications or diagnostic/testing equipment (elderly think that they are not supposed to get medications because their services are free)  Elderly with NHIF/CHF can get medications from pharmacy while others with no HI but have exemption/waiver cannot get (MOI-Ig, pg 4)  The identification process is not efficient because sometimes forms/pictures to get IDs are lost, names/pictures are mixed in the list and sometimes pictures are taken from the elderly repetitively without getting their IDs.  Sometime there is no special window/room with a doctor/medications for elderly.  Not all elderly have IDs.  Elderly with no any identification for their age it’s a challenge to get services from the health workers.  Elderly visited other districts or regions without IDs is unlikely to get services.  Services which were supposed to be covered by the elderly but have been provided by the LGAs at the camps are  Exemption on health issues  Clothes  Accommodation for the elderly  Poor elderly living in difficult condition  Food for the elderly  The criteria for the elderly to be taken to the elderly camps are  Poor elderly with no people who can support them  Elderly with no children who are in need  Elderly have been helped once they go to the health facility but the funds to finance their services it’s difficult to get from the government.  More than 30% of the elderly have IDs.  The elderly expectation are to have IDs through its process takes time.  Elderly matters/agenda depends with the audience once it’s presented, i.e. some take it positively and some take it negatively thinking that there are other things which are important than elderly matters (TSWO-Ig). | - Existence of elderly councils for discussing elderly matters - Existence of elderly problems (pp1) - Social welfare department act as link between council and elderly population - Common conditions facing elderly - Challenges facing identification process of the elderly - Criteria for the elderly to be taken to the elderly camps - Different perception about elderly agenda | - Existence of legally recognized councils for elderly population welfare - Social welfare office as a link to LGAs to elderly population - Challenges facing elderly - Elderly camps - Perceptions about elderly agenda. |  |
|  |  |  |  |  |  |
|  |  |  |  |  |  |
| 5 | Policy | - Exemption policy consider elderly with 60+ years old, which does not say about the ones who are poor - Exemption policy is only applicable to the public health facilities and not for private health facilities – DSWO - Social welfare office provides information about elderly – DHFF 3   Exemption policy (Its challenges)  According to policy, elderly are to be 100% exempted, but in practice, is not due to its procedures of exempting the elderly.  In the previous years, LGA used to provide CHF card to the elderly. This was because they were receiving matching fund from the central government which increased the LGA’s revenue  After the matching fund, came the loan policy of which IDs/letter from VEO were given to elderly as an identification to HF. –TMO  The policy does not categorize the elderly to be exempted, only if you are 60+ but they insist for those have HI either from the retired HI or have HI from their family members whether its NHIF or CHF to access health services at the HF. – TMO  There no compensation funds for the exemption from the elderly at the HF  Exemption does not mean is only for the elderly, it’s include other groups like pregnant women, <5 years children, TB patients, etc. In order to maintain the availability of drugs they use the available funds from the central government, own sources of funds, from unexampled clients, etc. | - Existence of exemption policy for the elderly matters for the public health facilities - Exemption/waiver to the elderly is automatic after 60+ *(Recommendations: Should not be used as the only criteria for waiver and it should be changed from the policy itself)* - No compensation funds for the exemption/waiver at the HF from the central government | - The burden and controversial of the exemption/waiver policy at the health facilities. |  |
|  |  |  |  |  |  |
| 6 | Systems | - The decentralization helps the priority setting process because during planning and setting budget involves all levels from lower level to the high level at district council level – DPLO 21 - DHFF focal person supervises the funds which goes direct to the health facility and report after receiving the bank statements from the TMO of the specific district council – DHFF 11 - There is no specific % of the budget for the elderly from the government or LGAs (TSWO-Ig) - The Government of Tanzania – Hospital Management Information System (GoT-HoMIS) helps to know - Medications dispensed - Medications which are out of store - Medications which are not in the store - The number of the elderly who were served per month - The types of elderly served and medications which are most consumed by the elderly - Before the current system (GoT-HoMIS) it was very difficult to know the exactly about exemption/waivers. This can be one of the mitigation on identifying the exactly cost of waivers/exemptions so as to be funded. | - Decentralized health system (LGAs) help coordination of planning for priority setting process at local level - DHFF helps to track funds from the central government to the HF - GoT-HoMIS helps to track funds collected at the HF - No specific percent of the budget for the elderly from LGAs | - Existence of Government systems at LGAs and HF helps to track provision of health services including elderly health care services - No specific percent of the budget for the elderly from LGAs |  |
|  |  |  |  |  |  |
|  |  |  |  |  |  |
| 7 | Stakeholders | - Social welfare educate the community through ward development committee (WDC) and other meetings - Most of the political parties including the ruling part protect elderly and other vulnerable groups like disabled people, etc. – DSWO 20 - SWO is the office which is responsible for the elderly by identifying them, and to give them IDs whereby the process starts from lower levels (village) – DPLO 6 - The functions of the DSWO; - To defend elderly’s interests/benefits - To deal with the directives from the central government about elderly - To provide IDs - To make sure that, medicine for the elderly are available at the health facility - To inspect the services provided to the elderly at the health facilities - To supervise the elderly councils each year and link them to the LGAs - Stakeholders include; - TASAF   Is not their role but they do help   - JIDA - MPs - Politicians - Community - There is no NGOs which are involved direct in elderly in the district, only few individuals who come and offer some funds or material things for the elderly (TSWO-Ig). | - Existence of stakeholders for representing/dealing elderly matters during setting priorities - Existence of the social welfare department to deal with elderly matters and supervise elderly councils - Existence of some of the NGOs are not much interested for the elderly health care services | - Health care services for the elderly (elderly matters) involve many stakeholders |  |
|  |  |  |  |  |  |
|  |  |  |  |  |  |
| 8 | Challenges of priority setting process | - The planning team has no special training about priority setting, use experience - Challenges during priority setting: - Scarce of resources (insufficient funds and human resources) - Difficult to identify elderly (age) and to get IDs - Difficult to predict the number of elderly per year (budget vs the number of elderly). - District councils are instructed by the central government to provide free health services to the elderly but no funds provided – Directives/Matamko (pg1 DSWO) - Availability of medicines for the elderly’s problems - Some of the Families do not take care of their elderly - Long waiting hours at the health facility - Low number of personnel (health workers) - The burden of the exemption to the health facility which still not known who covers the costs - DHFF have no any category which covers the costs of exemption – DHFF 7 - Ceiling budget is very low from the central government while all medications are very expensive. - No complaint about special window for the elderly from either elderly themselves or health workers – DHFF 23 - CHW are not well utilized to help elderly due to financial problems - Some health facilities don’t offer free services to the elderly (pg5) - CCHP indicates the percentage of each activity but not for the elderly – DPLO 4 - Regarding the transport from one hospital to another especially when the elderly have referral will depend on the seriousness of the disease but there is an ambulance which can help - DHFF 10 - No specific percentage of the budget for the elderly DPLO 3 - Complaints about elderly healthcare services in rural areas, some of the health facilities (HF) refuse to serve the elderly free, they are forced to pay even though they are eligible to be exempted (pg5) - Some HF mix elderly with ensured people but still prioritize elderly(pg5) - The challenge of the slogan of “pisha mzee atibiwe kwanza” is that, some of people in rural areas like politicians, rich people and famous are treated as elderly – DHFF 16 - Not all elderly have IDs – DHFF 14 - Exemption costs > Revenue (pg8) - Sometime the exemption gap vs revenue is filled by the basket funds (pg8) - There is no specific guideline which indicates the explanations about exemption for the elderly which can be followed by the health workers than statements and directives from the government officials (matamko kutoka kwa viongozi wa serikali) – DSWO - Most of the complaints from the health workers does not come direct due to waivers and exemptions rather than deficit of the budget of buying some of the things like uniforms which can be solved by finding more own sources of revenue – DPLO 16 - CHW?? Not responsible for elderly, they are responsible for children (under 5 years old) and mothers/pregnant women, although they can be assigned for the elderly on the outreach services (huduma ya mkoba) DPLO 29 - NGOs?? Most of the NGOs are not interested with elderly rather than other issues like AIDs, child health, pregnant women, etc. – DPLO 30 - MPs??? Are not much involved themselves on helping elderly, they are only involved on planning process – DPLO 31 - Elderly matters are not much prioritized at all levels include family level, village level, ward and district council level – DSWO - No specific budget for the elderly instead the budget is located for social protection under the social welfare department which include elderly (pg9) - There is no allocation of funds for the elderly per month or year but only depends on the activities to be done – DSWO - Results Based Financing (RBF) was used the CHW to solve some of the problems in the community especially the vulnerable groups include pregnant women, <5 years children, elderly, etc. (Outreach services) - The councils are not free as has been described that LGAs are autonomy, even the own sources of revenues councils should be given a ceiling budget or guideline from the central government – DPLO 22 - The burden of exemptions at district council level i.e. Exemption>revenue – DSWO 36 - Results based financing (RBF), basket funds, other charges (OC), etc. still low compared to exemptions – DSWO 38 - DHFF is just a system which does not touch direct the elderly, it’s just to allocate funds on each planned activity DHFF 4 - DHFF will only touch the elderly matters if the funded activity touches the elderly like providing funds for IDs – DHFF 5 - Challenges during priority setting includes; deficiency of resources (funds), infrastructure (special room for elderly), health workers at the health facility, un refundable funds (exemption), deficiency of medicines at the health facility – TMO   Limitation of LGAs for the priority setting process includes;  Deficiency of sources of revenue/resources from the LGAs  Own sources from the LGAs does not fund elderly health care services, they are funded from the central government-TMO   - LGAs does not prioritize health sector in general by thinking that, health sector at the facility level have funds.-TMO   The targeted number per year to provide 5000 but only 1000; so the process of providing IDs is not efficiency due budget deficit. For the CHF, there are only few elderly who join while others exit-TMO  There is no specific percentage of the budget which goes to the social welfare where the elderly belong. The budget is set according to situation analysis. -TMO  The implementation of different priorities are challenged with the budget deficit. -TMO  Collect small amount of out of pocket in many health facilities which is sometime zero. – TMO  Basket fund has been reduced and delay but services still provided to the patients  Most of health facility in their setting has no special window/room and low number of health workers.  Due to the exemption policy to the elderly has led to the increase of access to health care services among them, that’s why there is a special day for NCDs for the elderly for drug refilling (MOI-Ig)  Development Partners/stakeholders have their own interest and not elderly.  The burden of exemption/waivers (elderly, <5 years, pregnant women, poor people, some of diseases, etc.)  As the exemptions/waivers increase, the collected revenue decreases at the hospital, and in return decreases the availability of medications and other services.  The price of medications depends much on the level of treatment from level one, two, three, etc. i.e. as the level increases also the price of medications increase. | - Deficiency of well trained personnel of planning team for priority setting process - Insufficient funds to fulfill all prioritized elderly issues - Existence of moral issues of some of the family members about elderly - Difficult to identify elderly (age) from lower level for provision of IDs - Interference of the central government to the LGAs which seems to be autonomous - Some of the government systems/structure like CHW, DHFF, CCHP, RBF, etc. do not have a direct link to the elderly matters - Existence of the challenges of insufficient of the health worker at health facilities - Existence of the burden of exemption and waivers with no specific guideline for the elderly matters - Most of the stakeholders including families, MPs, NGOs, etc. are not much interested with elderly matters - Untimely reimbursement of funds from the central government for the planned budget at LGAs including funds for activities which touches elderly - Increase of the access to health services among the elderly due the exemption policy - Decrease of the availability of medications for the elderly | - Experts for planning and priority setting at the LGAs for the elderly matters - Scarcity of resources lead to unfulfilled prioritized elderly matters - Moral issues among family members about elderly - Elderly identification problem - Unintegrated health systems at LGAs which doesn’t help elderly as were intended - Politics interference on provision of health care services - The challenge of the exemption policy itself. |  |
| 9 | Recommendations | - The central government should release some of the sources of income to the LGAs so as to fulfill some of their priorities – DPLO 24 - Ways to improve priority setting process at LGAs are as follows - Councils should be autonomous as was supposed to be - Own sources of income should be returned to the LGAs - To let the LGAs to decide for their own (planning process should not been interfered by the central government) - Health workers still a problem - DPLO 33 - To increase the budget specifically for the elderly - To provide HI to the elderly - For the process of identifying and providing IDs can be done through outreach services - DSWO 20 - To have a list of the elderly at each village so as to simplify the elderly identification – DHFF 22 - To repay the exemption fees for the elderly by either the government or other stakeholders – DHFF 22 - To find the way of refilling the burden of exemptions at the health facility (the government authorities have their own priorities not exemptions) - Each activities at LGAs should include the elderly issues - These elderly councils Should be very strong to protect elderly’s interest and should be conducted frequently – DSWO - The elderly should be identified and given IDs to access healthcare services free which supposed to be their right because they have contributed a lot to their country since they were young –DPLO 15 - Own sources should consider the elderly (pg11) - At least 5% of the own sources should serve the elderly - To provide HI which will help the elderly to access health services including private health facilities – DSWO 22 - Deficit of the budget at district council levels has been caused by the return to the central government of the following tributes (DPLO 23); - Street venders levy/Ushuru wa machinga (machinga IDs) - Rent levy/Kodi ya majengo - Poster levy/Kodi ya mabango - IDs will only be sustainable if it will include the costs of the elderly to access health services – DSWO 38 - Before the own sources of income been taken by the central government it was possible to fulfill our priorities, there are internal auditors who can control the council expenditures DPLO 25 (central govt interference) - In order to have improved health services for the elderly there should be the following issues –DSWO 41 - To enact a law that will protect the elderly’s interests from family level to council level - There should a budget which is specific for the elderly healthcare services - The government through some institutes like TASAF, etc. should find a way of supporting direct the elderly living in poor conditions (single, no children, poor) - The availability of the medicines for the elderly - To provide HI to the elderly instead of IDs which does not help anything where there is a situation of unavailability of medics at the health facility and the elderly needs to go to the pharmacy - To find the way of refilling the burden of exemptions at the health facility (the government authorities have their own priorities not exemptions) - Each activities at LGAs should include the elderly issues - GoT-HOMIS is a system which help to record revenue and patients’ records which can simplify to calculate the collected revenue and exempted costs which seems to be higher than the collected revenue (pg8)   In order to avoid the challenges:  Polypharmacy  Integrate rooms but prioritizing elderly (the issue of health workers)  To insist elderly to have CHF cards  To educate the elderly if they have NHIF card to use them in order to access health services instead of not using them because they access free services at the health facility   - Health workers (awareness) to ask the elderly if they have NHIF or CHF card when they come at the health facility – TMO - Sustainability of provision of CHF is the availability of medications and other services at the health facility - Recommendations;   LGAs under DED should recognize that it’s their responsibility to plan and fund the health sector at the lower level   - The central government through MoH and Tamisemi should find a way on how to fund the exemptions   To supervise the revenue collection at the health facility, that is why nowadays has started to increase.  All health facilities within the council, have new system of Got-Homis which help to increase the revenue  To review the price of accessing health services. Later there was user fee which means you have to pay at the end of the services but now there is an introduction of cost sharing at each service which means that you have to pay at each services eg. X-ray payment, consultant fee, medications, etc.  Encourage development partners/stakeholders to have help elderly.  To plan a budget which will satisfy many activities set for the elderly health services.  The government should reimburse the planed budget to the LGAs on time.  To promote awareness to all members by explaining the importance and needs of the elderly agenda through different activities.  Identification process of the elderly should proceed and the budget should be allocated and reimbursed for the elderly services on time (TSWO-Ig)  Mitigation for the elderly complaints:  To follow guideline on how to dispense medications by following all steps and educate the health workers  To encourage elderly to have HI (those who able to enroll themselves, helped by their children/family members or those can be helped with other stakeholders like politicians).  To have enough budget for medications including the most consumed by the elderly | - Autonomous of the LGAs for planning process during setting priorities - Budget allocation for the elderly matters - Provision of HI instead of merely IDs - Improving ways of identifying elderly by using a database in each village/mtaa - To enact a law that will protect the elderly’s interest including financing process, health services, exemption policy, TASAF, etc. | - Autonomous of the LGAs for planning process during setting priorities - Improving allocation of resources for elderly matters - Improving health systems at LGAs to have improved elderly identification, provision of IDs/HI - Law for elderly interest protection on provision of health services | - Recommendations - Enact law specific to protect elderly interest - To have a specific % of the LGAs revenue which should go to the elderly health services (elderly matters) - To build capacity among stakeholders who are involved during planning and priority setting process (experts, decision makers, health workers, elderly councils, local leaders, community representatives, other stakeholders, etc. - Improving identification process by having a database at LGAs level which can be easy for identifying the number of elderly as an estimation for planning and priority setting - Sensitizing awareness among stakeholders including elderly, community, health workers, TASAF, NGOs, insisting elderly who have ability to have HI - Involving CHWs on identifying elderly and other elderly problems - To repay the exempted fees from either the central government or LGAs to the HF or provide HI |
| 10 | Community perception about elderly during priority setting | - Community perceptions about elderly, sometimes perceive them as witch crafts (wachawi) DPLO 27 - Some of the elderly are being helped at village/community level with the neighbors – DPLO 28 - Community involvement about elderly matters, i.e. helping elderly to reach the health facility where necessary, to provide information about the elderly i.e. contribute a little amount in the community for the elderly who are poor – DHFF 20 - Community participation on priority setting is the identification of the elderly which starts from the community through community meetings by helping the local leaders (hamlets, VEO, WEO to the district level) to provide information about the available elderly living with difficulties, and helps to promote democracy during identifying elderly because its more transparency. - Community perception: some people take consideration and some are not considering as elderly matters are important to consider (TSWO-Ig) - The community has been educated about elderly through the following: - Through World Elderly Day - Through village meetings - At the health facilities through morning sessions and through posters. - Through brochures (TSWO-Ig) - CHWs were not designed for <5 years and pregnant women but not for the elderly - It is difficult for the community to contribute for the elderly health care services because they knows that health care services for the elderly is free and the government is responsible - Society starts from individuals, it should start from family level, community, etc that everyone should be responsible to their parents/guardians. - Importance of elderly in the community for the vision and wisdom. | - Existence of different perceptions from the community about elderly - Existence of community involvement on elderly matters during priority setting - Community awareness about elderly | - Community perceptions about elderly - Community involvement during priority setting - Community awareness about elderly |  |
